# Image analysis of immediate full-arch prosthetic rehabilitations guided by a digital workflow: assessment of the discrepancy between planning and execution

**DOI:** 10.1186/s40729-019-0179-1

**Published:** 2019-07-15

**Authors:** Krzysztof Chmielewski, Wojciech Ryncarz, Orcan Yüksel, Pedro Goncalves, Kyung-won Baek, Susy Cok, Michel Dard

**Affiliations:** 1 SmileClinic Advanced Implant Center – Klinika Stomatologii Estetycznej i Implantologii, ul. Karola Szymanowskiego 2, 80-280 Gdańsk, Poland; 2 Stomatologia estetyczna implantologia – Klinika Proimplant, ul. Cecylii Śniegockiej 8, 00-430 Warszawa, Poland; 3 YÜKSEL | GIESENHAGEN Dentale Implantologie, Bockenheimer Landstr. 92, 60323 Frankfurt, Germany; 4 Institut Straumann AG, Peter Merian-Weg 12, 4052 Basel, Switzerland; 5 Oral, Diagnosis and Rehabilitation Sciences, College of Dental Medicine, Columbia University, 622 W. 168th St., New York, NY 10032 USA

**Keywords:** Digital workflow, CBCT, Full-arch rehabilitation, Virtual implant planning, Guided implant surgery

## Abstract

**Background:**

A dentition with adequate function and esthetics is essential for the well-being and quality of life. A full implant-retained fixed prosthetics is an ideal solution for fully edentulous arch, however requires complex planning, surgical, and prosthetic procedure. With the help of digital workflow, it becomes a predictable and fast solution for the dentists and the patients. This retrospective study analyzed the most advanced surgical approach in full-arch rehabilitation with dental implants and immediate loading using digital workflow.

**Methods:**

Patient records of fully edentulous jaws treated in four clinical centers in Warsaw, Poland, were evaluated. Computer-assisted planning and surgical template fabrication were done using the planning software coDiagnostiX™, based on a pre-op cone beam computed tomography (CBCT) and scanned data of a plaster model. A post-op CBCT was acquired after the placement of four to six implants by the guided system. The influence of different surgical variables on the discrepancy between planning and execution was analyzed, together with the biomechanical indices.

**Results:**

A total of nine patient records were selected of 12 edentulous jaws treated with 62 implants. The overall mean three-dimensional (3D) offset at the implant base was 1.60 mm, at the tip 1.86 mm. The mean angle of deviation was 4.89°, the mean implant stability quotient (ISQ) 70.42, and the insertion torque 35.58 Ncm. The 3D offsets were influenced by the gender of the patient, treated jaw, the diameter, and length of the implant. The angle of deviation was affected only by the treated jaw. Insertion torque was influenced by the treated jaw, the age of the patient, the length of the implant, tooth type, and the side of the jaw.

**Discussion:**

Bone quality of the patient and implant preparation procedure influenced the discrepancy between the planning and the execution of the digitally guided implant placement. Dense bone—mandible, posterior area, young age, and man—and multiple preparations of the implant bed—wider and longer implant—could be suggested as risk factors.

**Conclusion:**

Digital workflow successfully enabled the immediate full-arch rehabilitation with a predictable outcome by different surgeons in multiple centers.

## Introduction

Over the last hundred years, there have been compelling advancements in the restoration and replacement of missing teeth. The introduction of digital planning tools in dentistry has vastly changed traditional workflow, together with computer-aided design and computer-aided manufacturing (CAD/CAM) restorations [1]. Although the placement of dental implants has become part of routine dental procedure, digital planning and treatment are still new tools in implant dentistry. With these new tools, surgical techniques can be simplified to reduce patients’ discomfort [2, 3]. From the radiographic data acquired with cone beam computed tomography (CBCT), three-dimensional (3D) image models are reconstructed upon which dentists can plan the implant placement and the prosthesis design [4]. Each plan is based on the individual patient, considering the anatomical limitation and bone quality, assessed with the 3D models [5]. Guided surgery delivers this plan to the patient’s mouth. 3D implant planning software, linked to the guided surgery solution, is essential to this individualized surgical and prosthetic planning and execution.

Guided surgery can be static or dynamic [6]. With a static guidance, dentists place the implants using a mechanical surgical guide or template that indicates planned implant position through stepwise drilling procedures. The fabrication of the surgical guide or template can be done by digital CAD/CAM with stereolithographic or milling, or in a conventional way in dental laboratories. Dynamic guidance is also called image-guided surgery or computer-assisted navigation. The position of the surgical instrument is displayed on a monitor with respect to the 3D model of a patient and the corresponding digital planning. This enables the clinician to review in real time the discrepancy between the planned treatment and the current implant placement. On the other hand, static guidance does not permit changes made at the time of surgery, as the sleeves of the surgical template grant a high degree of controlled drilling. Even though dynamic guidance fulfills more the concept of digital planning and treatment, currently proven accuracy is higher with static guidance [7]. This is mainly due to the inaccuracy of the 3D-3D registration of the patient’s 3D model with the actual patient, which is a necessity only in dynamic guidance. Static guidance is also the established digital workflow of the majority of dental implant systems.

Despite advances in preventive dentistry, edentulism is still an increasing major public health problem [8]. Fully edentulous patients often have problems with the prostheses with poor retention, because of their absorbed bone and weakened neuromuscular control [9]. An implant-supported overdenture or a fixed full denture can mitigate this problem significantly [10, 11]. Here, to make the best use of their remaining bone and avoid the augmentation as much as possible, digital implant planning plays an important role. 3D images allow bone quality and anatomical considerations incorporated in planning the prosthesis. Guided surgery with a surgical template enables minimally invasive implant placement, results in less pain and swelling. Considering more than 90% of fully edentulous patients need an implant-supported prosthesis, the role of digital implant planning and treatment is crucial to provide these patients more functional, comfortable, and esthetic prosthetic solutions.

Several digital planning tools and guided surgery solutions exist in the market, but their concepts and the results are at variance. When it comes to the treatment of fully edentulous arch, the difference of the treatment concept becomes prominent, regarding the preservation and conditioning of the remaining bone. Pro Arch is a new treatment concept of full-arch rehabilitation, with immediacy and individualized treatment plan. Implant placement and prosthesis design are tailored to each patient so it covers various treatment options, unlike a well-established treatment concept of all-on-four. Combination of this concept and digital workflow, we can provide the most advanced surgical approach to fully edentulous patients. This retrospective case study analyzed the discrepancy between the planned implant position and the final implant insertion from 12 jaws treated by Pro Arch concept in multiple centers. Furthermore, biomechanical assessments of the implant insertion torque and implant stability quotient (ISQ) were included in the analysis. We aimed to identify the risk factors influencing the discrepancy and suggest how to overcome these risk factors to make improvements in implant placement. The authors hypothesized that the discrepancy of the planned and actually placed implant position is within the range of few published data. Moreover, we anticipated that certain major risk factors would be identified for full-arch restoration with a digital workflow.

## Materials and methods

### Study design

This study consists of a collection of cases selected from the medical records of four clinics in Warsaw, Poland. Initially, the records of patients with fully edentulous jaws were pooled according to the willingness of the patients to receive an immediately functioning full-arch prosthesis within 24 h after implant surgery. The group of four surgeons identified retrospectively the suitable cases to be assessed based on the outcomes of consensus meetings where they shared their respective treatment and imaging analysis protocols and challenged them against the state of the art. These records were evaluated for adequacy in April 2016, to ensure that the clinical cases showed a consistent treatment and analysis reproducibility. At the end of this process, nine patient records were retained.

### Subject population

Nine patient records from four clinical centers in Warsaw, Poland, were selected in a consecutive manner according to the following criteria; complete edentulous males or females with the age of 18 or more, who voluntarily had signed an informed consent form to allow for use of their medical records for study purpose. The exclusion criteria were classified as systemic, local, and intra-operative. The systemic exclusion criteria were systemic diseases that would interfere with dental implant therapy, smokers of more than ten cigarettes a day, alcoholism or drug abuse, subjects with inadequate oral hygiene, subjects who have undergone administration of any investigational device within 30 days prior to the enrolment in the study, physical or mental disabilities, pregnant or breastfeeding women and conditions or circumstances that would prevent completion of study participation or interfere with analysis of study results, such as history of non-compliance or unreliability. The local exclusion criteria were any bone augmentation procedure on the implant site, local inflammation**,** mucosal disease, history of local irradiation therapy in the head and neck area, and extraction sockets with less than 8 weeks of healing. The only intra-operative exclusion criterion was the lack of primary stability of the dental implant.

### Diagnostic and planning procedures

Following the conventional prosthetic procedures, all subjects received a new complete denture, which was tried and confirmed in advance to meet the agreed standards of esthetics and function. In order to set a radiopaque reference for a planning tool, small holes were drilled on the occlusal surface of the posterior teeth and the cingulum of the anterior teeth of this barium sulfate acrylic denture and filled with flowable resin composite. The marked denture was placed in the patient’s mouth for the CBCT, with open bite position using cotton rolls between the teeth. The radiographic images were acquired as uncompressed Digital Imaging and Communications in Medicine (DICOM) file.

Two to 3 weeks before the implant placement, three provisional implants of Ø 2.5 mm diameter and 8.5 mm length (MiNi Overdenture, MegaGen, Korea) were placed to secure a stable and precise position for the surgical template. After this, a pick-up impression with A-Silicone (Elite HD+, Zhermack, Italy) was taken in a closed tray to prepare a plaster model with the corresponding lab analogs. This model was scanned with an optical lab scanner (Straumann® CARES® 3 series, Institut Straumann AG, Switzerland) to obtain a stereolithography (STL) file.

Both STL and DICOM files were imported and meshed into the treatment planning software (coDiagnostiX™, Dental Wings, Canada). The digital planning of the implant position and the selection of the abutments were performed using coDiagnostiX. As for this, the ridge width and height were evaluated and areas with a width of at least 6 mm were selected. Each subject was planned to receive four to six dental implants (Straumann® Bone Level Tapered [BLT] Roxolid® SLActive®, Institut Straumann AG, Switzerland) per arch, which were positioned at least 3 mm away from the provisional ones. A static surgical template was printed out (3D medical print KG, Austria) according to the digital planning, also using coDiagnostiX. This template had serial drill guides (T-Sleeve, Institut Straumann AG, Switzerland) and reference marks to help the final position of the implant. Before the surgical procedure, the template was fixed in the patient’s mouth over the provisional implants and the mucosa.

### Surgical and prosthetic procedures

Prior to the start of the surgery, patients rinsed the mouth with 0.15 ml of 0.2% chlorhexidine for 60 s and 4% articaine with 1:100,000 epinephrine was injected on the surgical site. Sixty-two implants were placed according to the surgical template in 12 edentulous jaws by six experienced surgeons. Straumann® BLT implants with a diameter of Ø 3.3 mm (NC) or Ø 4.1 mm (RC), and with a length between 10 and 14 mm, were placed following the recommended drill sequences (Straumann® Guided Surgery Cassette, Institut Straumann AG, Switzerland). Implants with 8 mm length were placed only when the bone height was limited or where the 5th or 6th implant was necessary in the posterior mandible. Where the space was limited, the insertion path of the implant collided with existing provisional implants. In such cases, two surgical templates were prepared; one for placing the implants that are not colliding with the provisional implants and the second one for the colliding implants to be used after removing the provisional implants. The surgery was done either flapless or with a minimally invasive mucoperiosteal flap. The bone type was assessed by the clinician, according to its density during the drilling and implant placement. Each stage of drilling was copiously irrigated with sterile sodium chloride, and all implants were placed in accordance with the 3D planning. Implant motor with documentation function (Elcomed, W&H GmbH, Austria) was used for the torque insertion registration and Osstell IDx (Osstell AB, Sweden) for the resonance frequency analysis, which is shown in ISQ-scale. These measurements were recorded and stored digitally.

Immediately after the surgery, pre-selected screw-retained abutments were fitted to the implants with a definitive torque of 35 Ncm and non-engaging titanium copings (Institut Straumann AG, Switzerland) were hand tightened into all of them. The screw access holes were covered with sterile polytetrafluoroethylene (PTFE) tape in order to prevent the entry of any external material. After creating holes over the titanium copings, the new complete denture was placed in the patient’s mouth to confirm that it could be re-seated on the provisional implants without interference. This position over the provisional implants served as a baseline for the originally planned occlusion and the proper stabilization when the prosthesis was relined and re-attached. After the confirmation, removed holes of the denture were filled with cold acrylic (PalaXpress, Kulzer, Germany) and placed back into the mouth, with the patient gently biting on it. After the acrylic was set around the titanium coping, all screws were untightened to remove the denture to trim the excess and sharp edges. Once the trimming was finished, the denture was screwed in the mouth with an insertion torque of 15 Ncm. The access holes were filled with sterile PTFE tape and the screw complex was sealed with composite. Finally, the denture was polished with the polishing tools (Brasseler GmbH, Germany). At the end of the procedure, the final CBCT scan was acquired to control the marginal bone levels and to evaluate the accuracy of the guided surgery. The same scanner setting was used as with the primary scan for the implant planning.

### 3D analysis

**Fig. 1:**
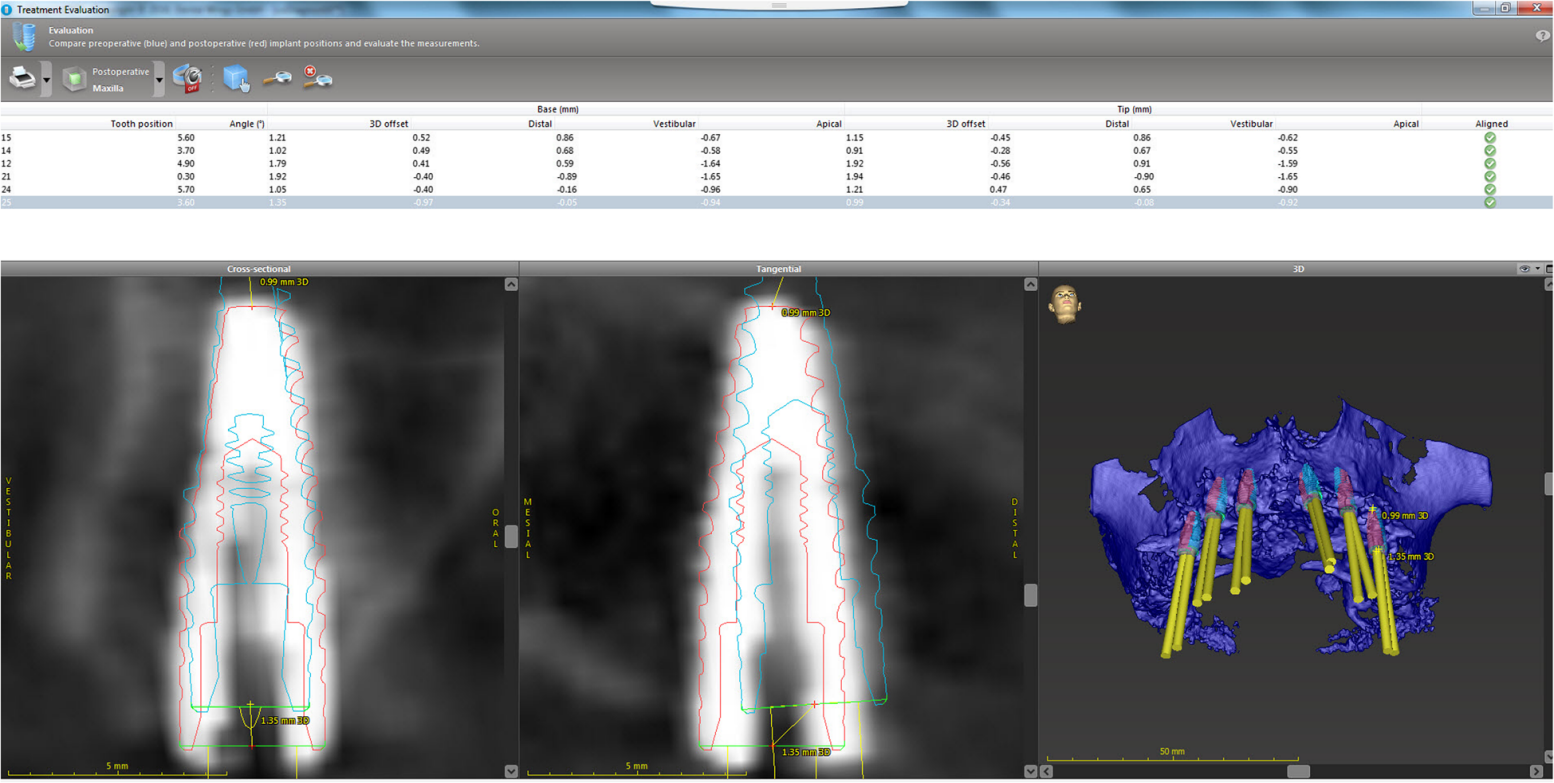
Screen capture of the software-based 3D analysis of the implants placed in the Maxilla. Lower figures are the comparison of the planned position (blue contour line) and the actual insertion (red contour line) of #25i

Following the software-based treatment evaluation tool with automatic image fusion, the images of the actually placed implants were superimposed over those of the planned ones. These images were highlighted by red and blue contour lines, respectively. The implant positions were automatically detected along the *x*, *y*, and *z* axes. The *z* axis was determined by the axis of the planned implant and detecting the superior-inferior deviations. The *y* axis was perpendicular to the *z* axis and therefore showed horizontal deviations in the oro-vestibular direction. The *x* axis was perpendicular to the *y* and *z* axes and detected horizontal deviations in the mesio-distal direction. The total deviation in space (3D deviation) and the deviation between the axes were detected at the level of the implant shoulder (base) and the implant apex (tip). In the end, the following three variables were assessed to evaluate the discrepancy; base 3D offset, tip 3D offset, and angle of deviation (Fig. 1).

### Statistical analysis

To identify the risk factors, the influence of the surgical variables (surgeon, jaw type, side, implant position, length, and diameter of the implant) on the discrepancy of the planned and placed implant positions was evaluated (“Part I” section). Additionally, biomechanical assessment of the implant insertion torque and ISQ was included in the evaluation, related to the surgical variables (“Part II” section). The below mentioned methodology was reviewed by the biostatistician from the university.

Parameters of categorical nature were summarized as counts and proportions, continuous parameters as means, standard deviations, medians, and interquartile ranges. To examine the effect of potential influencing factors on implant placement, the association between the accuracy parameters and each of the probable factors was examined first. This is, mixed linear regression models with only one predictor but adjusted by the effect of an individual (introduced in the model as a random effect) were performed. The associations between implant accuracy placement parameters and all of the potential factors, which could have an influence, were examined using multivariable mixed linear regression models. The surgeon, the number of implants per prosthesis, the patient’s age and gender, the patient smoking, the diameter and length of the implant, the tooth type, and the side of the jaw were included in the model as fix effects. The effect of the patient was included as a random effect. The backward selection was used to reduce the models, and factors with a *p* > 0.2 were excluded. The model performance was checked using the Bayesian information criteria after each exclusion. Patient’s age was the only factor excluded from the model. The effect of smoking could not be determined since there was only one current smoker. The Dunnett-Hsu adjustment was used to correct *p* values for multiple comparisons. The significance level was set to an alpha < 0.05. SAS version 9.4 (2002-2012, SAS Institute Inc., Cary, NC, USA) was used to perform the statistical analysis.

## Results

A total of nine patients were treated in 12 edentulous jaws with 62 implants, in four centers by six surgeons. Patients’ characteristics are described in Table 1. The overall mean 3D offset at the implant base was 1.60 ± 0.86 mm and at the tip 1.86 ± 1.09 mm. The overall mean angle of deviation was 4.89 ± 2.50°. The overall mean ISQ was 70.42 ± 8.10 and the insertion torque 35.58 ± 15.99 Ncm. The descriptive statistics of the implant accuracy placement parameters and the implant biomechanical parameters are shown in Tables 2 and 3.

**Table 1:** Patients’ characteristics (*n* = 9)

Characteristic	Parameter	Value
Gender		
Female	*n* (%)	3 (33.3)
Male		6 (66.7)
Smoking		
No	*n* (%)	8 (88.9)
Yes		1 (11.1)
Age (years)	*n*	9
All patients	Mean ± SD	70 ± 11.5
	Median (IQR)	64 (63 to 81)
Age (years)	*n*	3
Female patients	Mean ± SD	76.7 ± 10.2
	Median (IQR)	80 (64 to 83)
Age (years)	*n*	6
Male patients	Mean ± SD	67.2 ± 11.9
	Median (IQR)	63 (58 to 81)
Prostheses type		
Maxilla + mandible	*n*	3 men
Maxilla		1 woman
Mandible		3 men + 2 women

*SD* standard deviation, *IQR* interquartile range (from the first quartile to the third quartile)

**Table 2:** Descriptive statistics for the different outcomes of the implant accuracy placement parameters (*n* = 62 implants)

Outcome	Parameter	Value
Base 3D offset [mm]	Mean ± SD	1.60 ± 0.86
	Median (IQR)	1.62 (1.03 to 1.92)
Tip 3D offset [mm]	Mean ± SD	1.86 ± 1.086
	Median (IQR)	1.575 (1.15 to 2.49)
Angle of deviation [°]	Mean ± SD	4.89 ± 2.50
	Median (IQR)	4.9 (3.3 to 6.6)

*SD* standard deviation, *IQR* interquartile range (from the first quartile to the third quartile)

**Table 3:** Descriptive statistics for the different outcomes of the implant biomechanical parameters (*n* = 62 implants)

Outcome	Parameter	Value
Implant stability quotient	Mean ± SD	70.42 ± 8.10
	Median (IQR)	72 (68 to 75)
Insertion torque [Ncm]	Mean ± SD	35.58 ± 15.99
	Median (IQR)	35 (20 to 50)

*SD* standard deviation, *IQR* interquartile range (from the first quartile to the third quartile)

### Part I

**Fig. 2:**
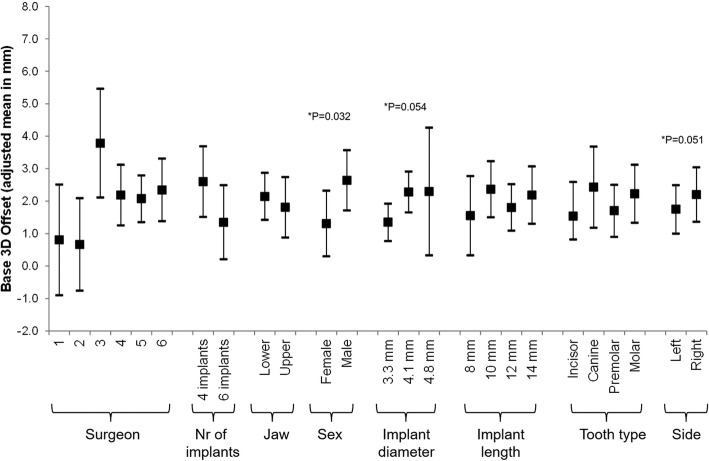
Multivariable association between Base 3D Offset and all considered risk factors. A mixed regression model was used. The effect of the patient was introduced in the model as a random effect

**Fig. 3:**
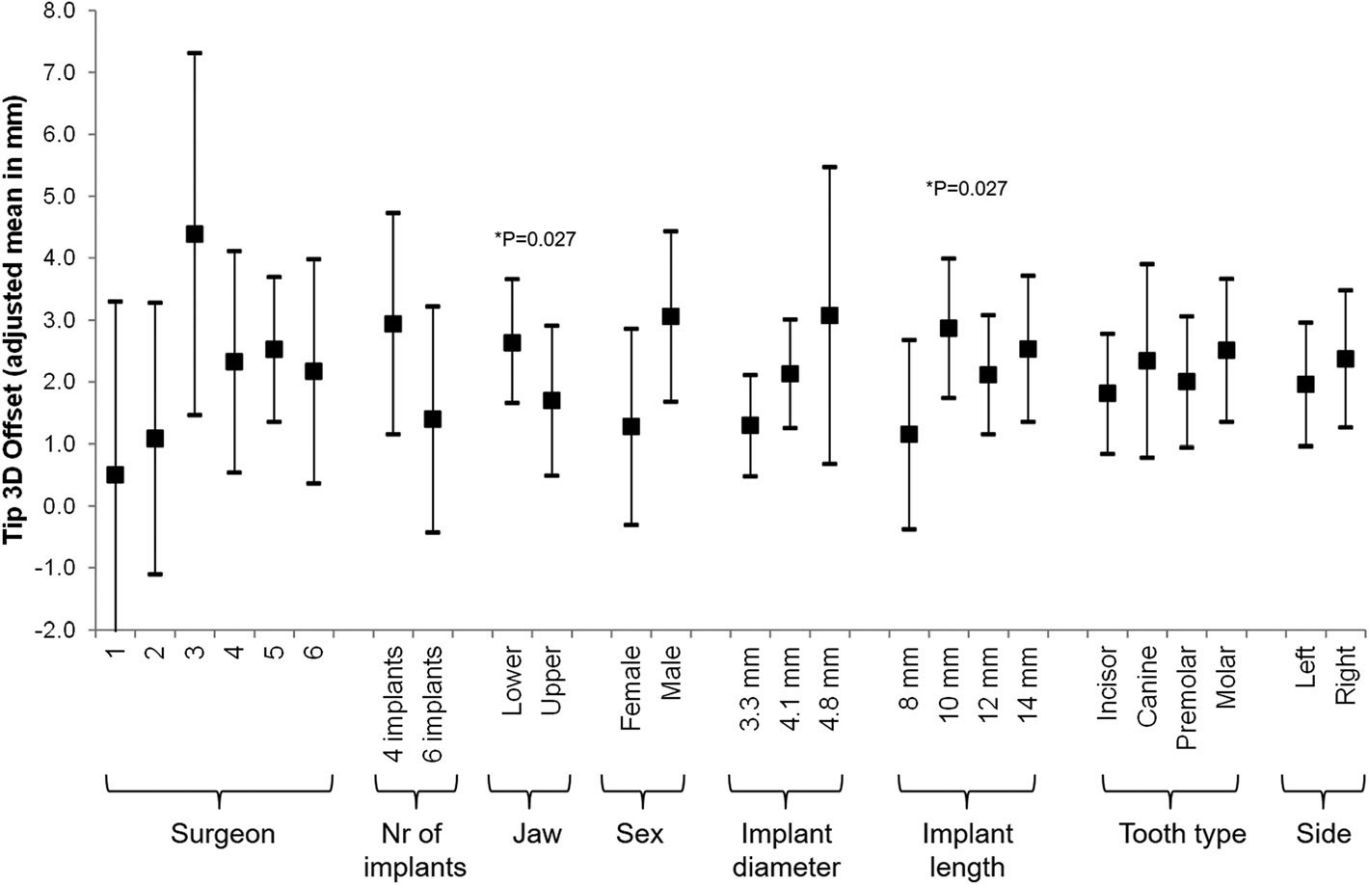
Multivariable association between Tip 3D Offset and all considered risk factors. A mixed regression model was used. The effect of the patient was introduced in the model as a random effect

**Fig. 4:**
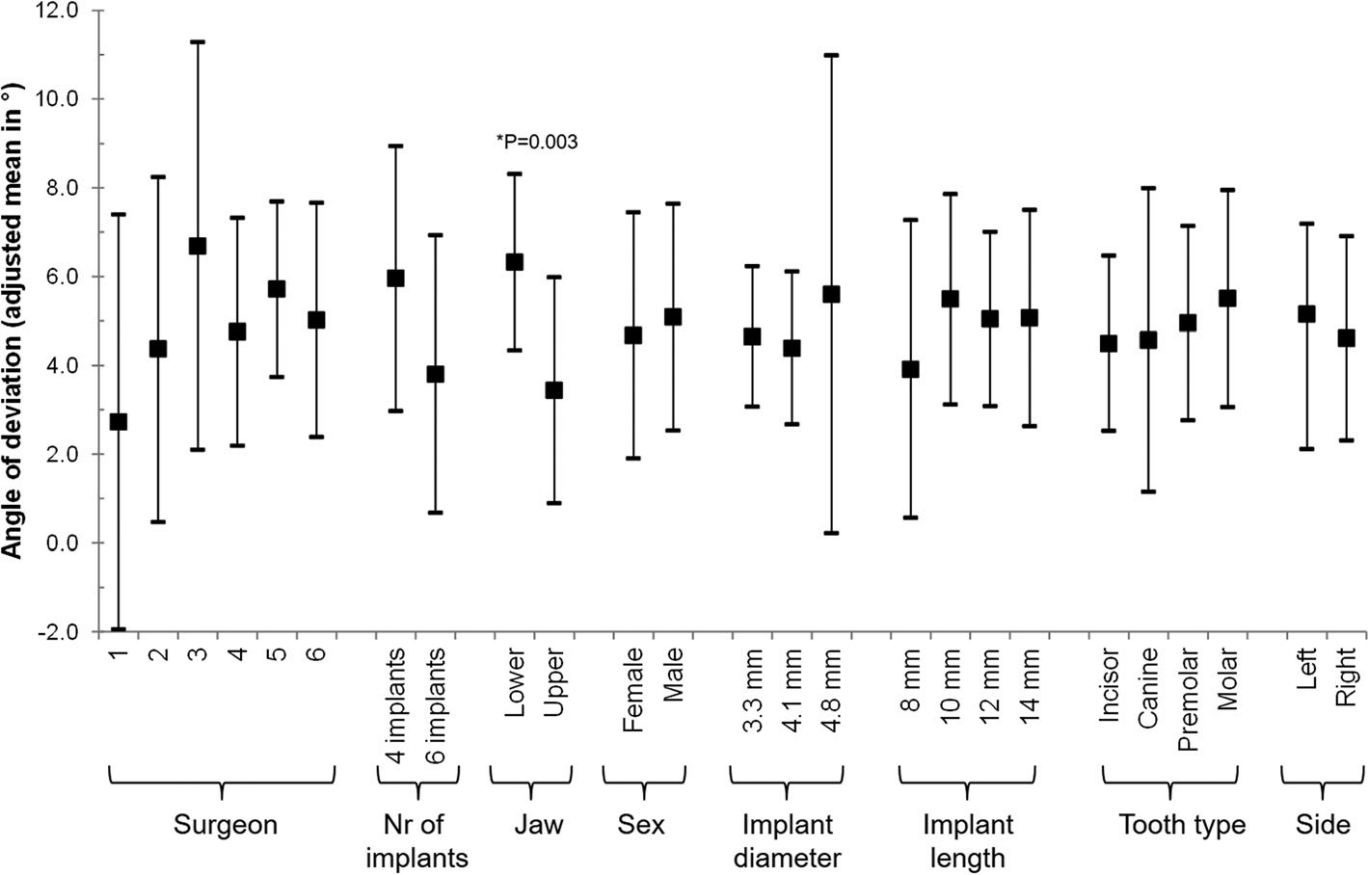
Multivariable association between angle of deviation and all considered risk factors. A mixed regression model was used. The effect of the patient was introduced in the model as a random effect

Multivariable analysis revealed that the factor surgeons, number of implants per jaw, and tooth type did not have an effect on the discrepancy between planned implant position and actual implant placement. Base 3D offset was influenced by the gender of the patient (lower offset for woman [adjusted mean (95% CI): 1.31 (0.30–2.32) vs. 2.64 (1.71–3.57) in men]), the diameter of the implant (lower in smaller [1.35(0.77–1.93), 2.28 (1.65–2.91), and 2.29 (0.33–4.26) for 3.3 mm, 4.1 mm, and 4.8 mm]), and possibly the side of the treated jaw (lower in the left side [1.75 (1.01–2.49) vs. 2.20 (1.36–3.04) in the right side]) (Fig. 2). Tip 3D offset was influenced by the treated jaw (lower offset in the upper jaw [1.70 (1.66–3.61) vs. 2.63 (0.49–2.91) in the lower jaw]), and the length of the implant (lower offsets in shorter implants [1.15 (− 0.38–2.68), 2.87 (1.74–4.00), 2.11 (1.52–3.08), and 2.53 (1.35–3.72) for 8 mm, 10 mm, 12 mm, and 14 mm]). The effect of the diameter of the implant was not statistically significant, but one could observe that the offset increases with an increase in diameter (Fig. 3). The angle of deviation was affected only by the treated jaw (lower deviation in the upper jaws [3.43(0.89–5.98) vs. 6.32(4.34–8.31) in the lower jaw]) (Fig. 4).

### Part II

**Fig. 5:**
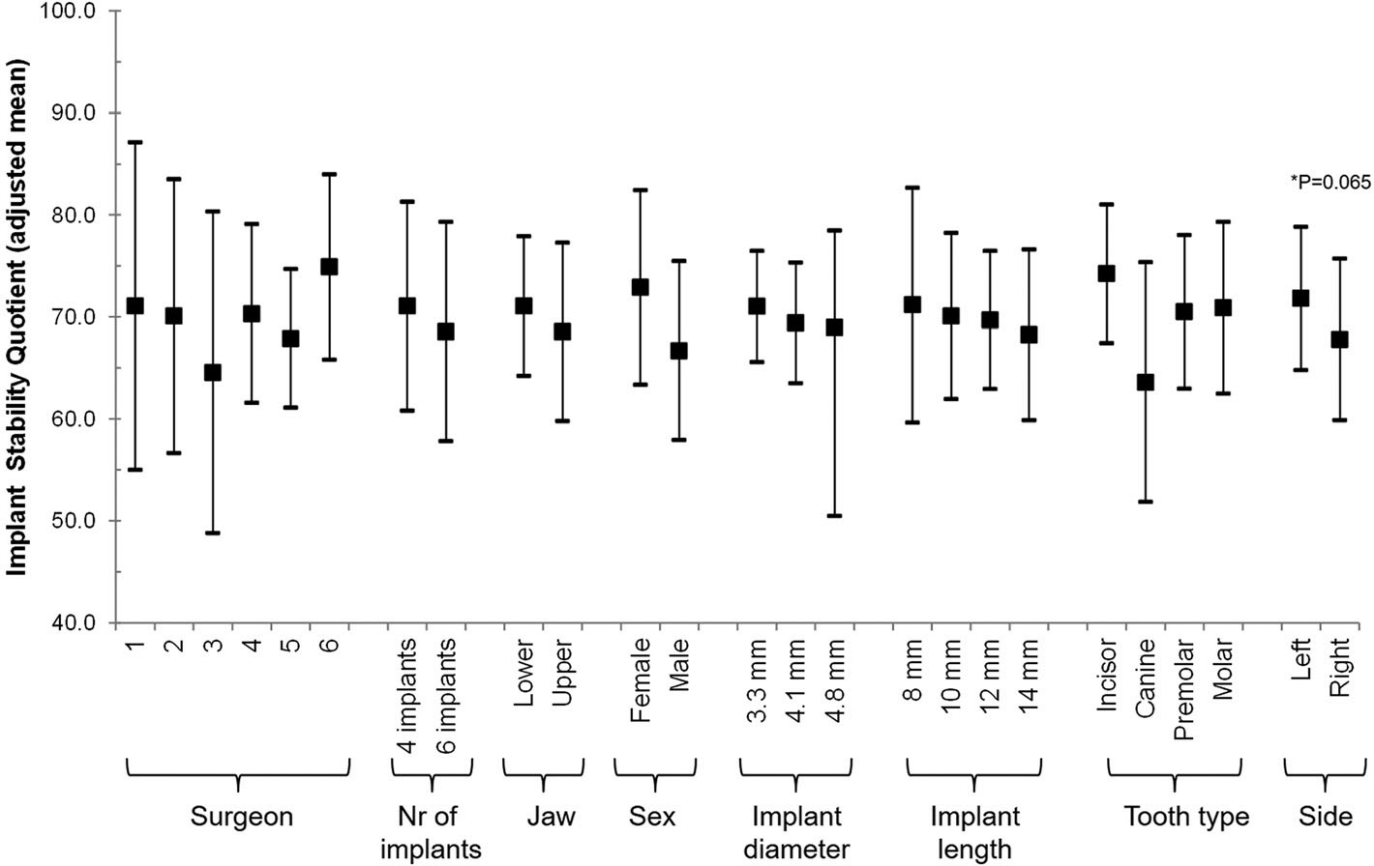
Multivariable association between ISQ and all considered risk factors. A mixed regression model was used. The effect of the patient was introduced in the model as a random effect

**Fig. 6:**
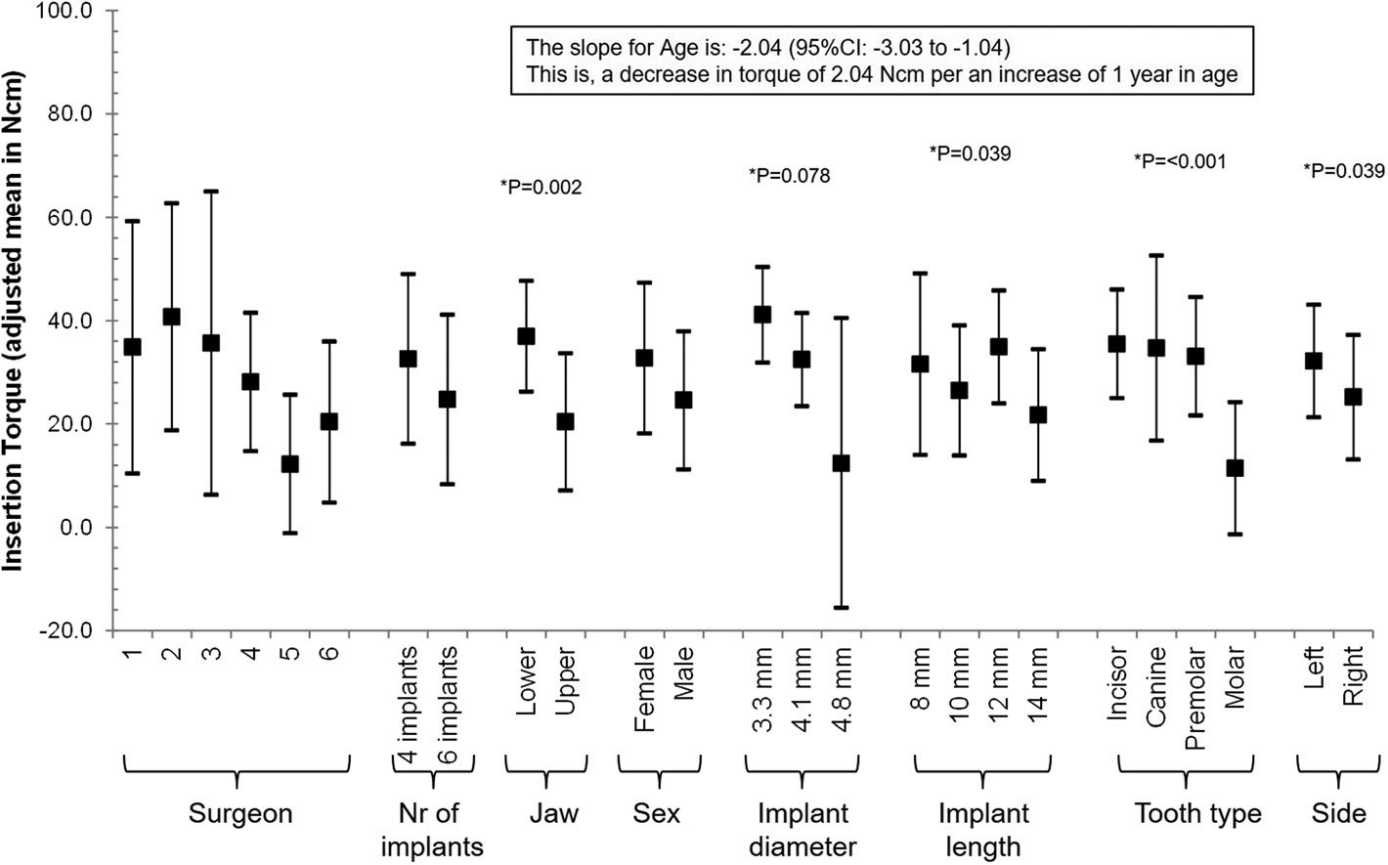
Multivariable association between insertion torque and all considered risk factors, including Age as a risk factor. A mixed regression model was used. The effect of the patient was introduced in the model as a random effect

**Fig. 7:**
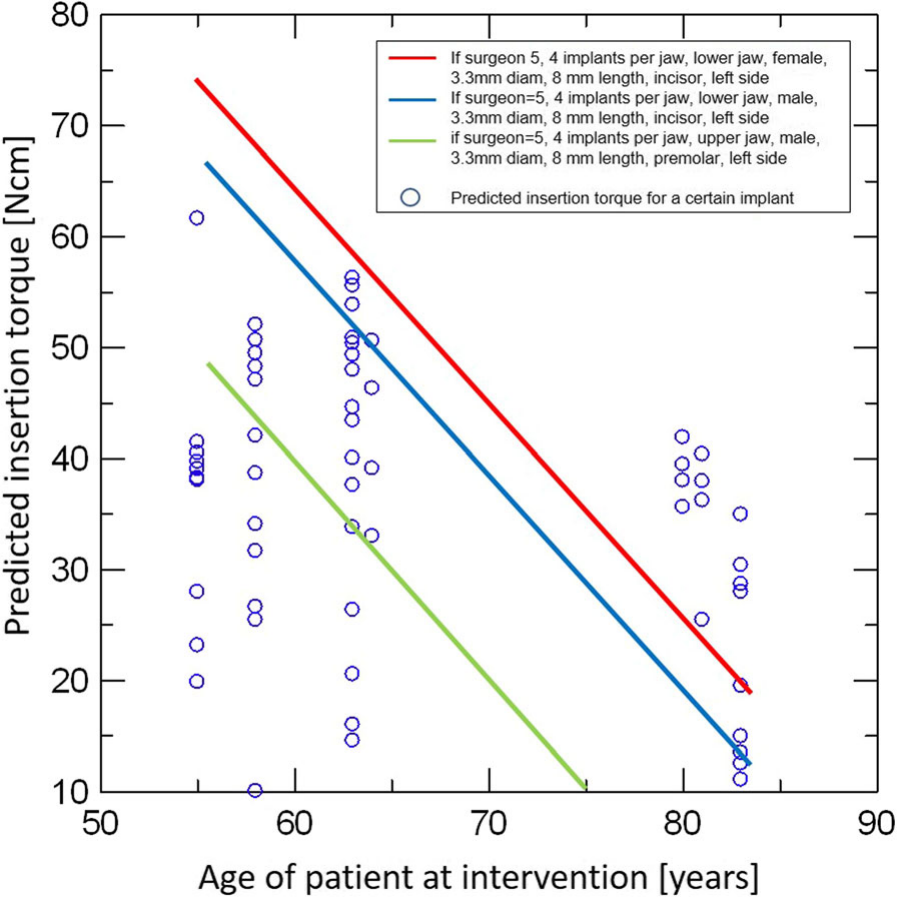
Predicted insertion torque and the age of the patient at intervention. To be able to plot predicted insertion torque versus age, the predicted insertion torque was calculated at a certain value of each parameter in the same model

Multivariable mixed regression models showed that ISQ might be influenced only by the side of the jaw (lower in right side [adjusted mean (95% CI): 67.78 (59.88–75.69) vs. 71.80 (64.79–78,381) in the left side]), but it was not statistically significant (Fig. 5). Insertion torque was influenced by the treated jaw (lower in the upper jaw [20.38 (7.12–33.64) vs. 36.95 (26.19–47.74) in the lower jaw]), the age of the patient (decreases 2.040 Ncm with an increase of 1 year of age [*p* value = 0.0002]), the length of the implant (highest in 12 mm [34.91 (23.96–45.85) vs. 31.56 (14.02–49.11), 26.49 (13.92–39.05), and 21.71 (8.98–34.43) in 8 mm, 10 mm, and 14 mm]), tooth type (lower in the molar [11.44 (− 1.39–24.28) vs. 35.47 (24.94–46.00), 34.68 (17.79–52.58), and 33.06 (21.57–44.55) in incisor, canine, and premolar]) and the side of the jaw (lower in the right side [25.18 vs. 32.15 in the left side]; Fig. 6). The effect of age on the predicted insertion torque is shown in Fig. 7, where we can see the insertion torque decreases with increasing patient’s age.

## Discussion

There is a certain number of studies which analyzed the accuracy, mostly in terms of the discrepancy between the planning and the execution, of the digitally guided implant placement. However, as there is no agreed definition of the digital workflow yet, different digital procedures were involved in the study conditions and their results were analyzed in various ways. Still, we could find that all these studies shared a similar aim, to prove the effectiveness of the digital workflow, and to suggest a way to improve it. Similar results could be drawn spite of their inhomogeneous study setups.

Several studies evaluated guided implant placement in comparison to the conventional procedure. Two in vitro (on acrylic model) studies compared guided drilling and free-hand drilling [12, 13]. In single-tooth gaps, they showed 1–1.5 mm of horizontal deviation and 1 mm or more of vertical deviation in free-hand drilling; however, no comparison was possible in partially and completely edentulous jaws. In an interesting in vitro study with inexperienced clinicians, Toyoshima et al. compared the planning and actual placement of a single implant with a surgical guide (static guidance) using coDiagnostiX [14]. From the superimposition of preoperative and postoperative CT images, they calculated three values—angle displacement of the implant axis, the 3D offset of implant base, and tip—to find less deviation at mandibular premolar area than molar area. Zhao et al. showed the same result of higher deviation in the posterior area than the anterior area, from 11 patients [15]. In a 2-year randomized clinical trial, Amorfini et al. compared immediately loaded implants of the maxilla by guided surgery to the standard procedure [16]. The guided surgery group showed statistically significant higher precision and the reduction of chair side time, invasiveness, post-op swelling and pain, and patient-perceived safety. However, overall clinical performance and global satisfaction scores were similar in both groups, so the authors concluded that the guided surgery might be reserved for more demanding cases. All the studies referred above were done in the partially edentulous jaws. It is important to understand, therefore, that our study condition of full edentulous jaws makes the cases more complicated and demanding.

Some studies evaluated the accuracy and discrepancy of the surgical template itself in vitro. Commonly, they showed higher accuracy compared to the clinical outcome of the actual implantation. In Kühl et al.’s study, the technical accuracy of printed surgical template showed a discrepancy of 0.22 mm in the sleeve top, 0.24 mm in the sleeve base, and 1.5° in angular deviation [17]. In Neumiester, Schulz, and Glodecki’s study, angle deviation was even lower (0.25°) with the 3D-printed drill template made by coDiagnostiX [18]. Both studies used coDiagnostiX for measurements. Bigger discrepancies in the clinical outcome come from the multiple clinical variances, but a big issue would be how the surgical template is placed in the patient’s mouth. Zhao et al. compared different surgical template fixations in 11 patients and concluded that the tooth-supported templates showed the higher accuracy than mucosa-supported ones [15]. Geng et al. compared the accuracy of different CAD/CAM systems and drew the same conclusion from 24 patients [19]. In a systematic review and meta-analysis, Gallardo et al. also concluded that the tooth support gives the highest accuracy [20]. An interesting finding in their study was that the bone support showed the biggest discrepancy, worse than the mucosa support. In fully edentulous jaws, like our study condition, a secure placement of the surgical template is crucial and at the same time challenging. Therefore, we placed three provisional implants before the surgery and used them to give additional support to the surgical templates. We could call it as a modified mucosa and tooth-supported guide, which should give the most secure placement up to date so far.

Other studies focused on the comparison of different guided surgery systems. In a systematic review of computer-guided template (static guidance)-based implant dentistry, Schneider et al. evaluated four parameters for accuracy—deviation at entry point (base), deviation at apex (tip), deviation in height, and deviation of the axis (angle)—and analyzed the clinical performance of 10 prospective clinical studies [21]. The deviation of the overall studies (1 mm at the base, 1.6 mm at the tip, 0.5 mm in height, and 5–6° in the angle) was similar to our study (1.6 mm at the base, 1.86 mm at the tip, 4.89° in the angle). They concluded that the clinical performance and the survival rate of implants placed with computer-guided technology were comparable to conventionally placed implants. However, there was a large variation among the studies, and the authors suggested that computer-guided surgery is insufficient to justify a “blind” implantation, especially in the flapless procedure. Two clinical studies included in this systematic review used the same software as our study (coDiagnostiX) for the analysis. Mischkowski et al. compared static guidance to dynamic guidance, using three computer-assisted fabricated surgical templates, including coDiagnostiX, and two image-guided navigations. Based on the successful application of all methods and easier handling, they concluded that the static template technique can be recommended for complicated implantology [7]. Nickenig and Eitner assessed 102 patients treated with virtual implant planning and surgical guide template, including 58.1% of flapless surgery, and concluded it to be a reliable procedure [22]. Naziri, Schramm, and Wilde assessed the accuracy of five different computer-assisted and template-guided implant systems, using coDiagnostiX [23]. With a similar analysis to our study, their result showed significantly lower deviation in a single-tooth implant than in a free-end arch. Similarly, longer implants resulted in a bigger deviation. These results from different studies seem to be aligned to our result that shorter implants and smaller diameter (anterior area) implants result in less deviation. As our study was conducted with fully edentulous patients, higher deviation of our result than several studies can be explained based on these studies. Likewise, using the guided surgery in our study can be also justified from their conclusion.

An interesting finding from our study was the reverse association of the patient’s age and the implant insertion torque. With a strong statistical significance (*p* = 0.0002), the insertion torque decreases 2.040 Ncm with an increase of 1 year of patient’s age (Fig. 6). Considering other variables related to the low insertion torque together, like the upper jaw and posterior area, we speculated this age factor could represent the effect of bone quality on the implant insertion. Based on the same presumption, Herekar et al. suggested their own scoring system to assess the bone density and implant primary stability [24]. BITS score is proposed to establish a correlation among the bone density values from CT, maximum insertion torque values, and resonance frequency analysis of dental implants. In this study, using 60 implant sites, the difference between insertion torque values and the bone type was found to be statistically not significant. On the other hand, in a human clinical and cadaver study more focused on the radiologic evaluation of bone quality by CT, Turkyilmaz et al. showed a strong correlation among CT-measured bone density, implant fastening (insertion) torque value, and ISQ values [25, 26]. In a biomechanical assessment with 298 patients, Alsaadi et al. drew a similar conclusion that the radiologically assessed bone quality was related to the insertion torque and ISQ [27]. In our study, we assumed that the decrease of the insertion torque with the increase of age showed the decrease of bone quality. A similar assumption was made with the lower insertion torque in the maxilla, the posterior area, and (aged) woman. Putting our results from the “Part I” and “Part II” sections together, we concluded that the bone quality of the patient (treated jaw, tooth type, and patient’s age and gender) and implant preparation procedure (implant diameter, implant length) influence the discrepancy between the planning and the execution of the digitally guided implant placement. Dense bone—mandible, posterior area, young age, and man—and multi-step preparation of the implant bed—wider and longer implants—could be suggested as risk factors. As this is not so much different situation for the conventional workflow, a thorough understanding of the patient’s condition and considerate approach is required to overcome these risk factors and improve digital workflow in implant placements.

## Conclusion

Digital workflow enabled immediate full-arch prosthetic rehabilitation by different surgeons in multiple centers. Bone quality of the patient and implant preparation procedure influenced the discrepancy between the planning and the execution of the digitally guided implant placement. Dense bone—mandible, posterior area, young age, and man—and multiple preparations of the implant bed—wider and longer implant—could be suggested as risk factors. As well as with conventional workflow, a thorough understanding of the patient’s condition and the procedure is prerequisite for the successful implant placement with a digital workflow.

## Abbreviations

3D: Three-dimensional
CAD/CAM: Computer-aided design and computer-aided manufacturing
CBCT: Cone beam computed tomography
DICOM: Digital imaging and communications in medicine
ISQ: Implant stability quotient
STL: Stereolithography
